# DustSCAN: A Five Year (2018-2022) Hourly Dataset of Dust Plumes From SEVIRI

**DOI:** 10.1038/s41597-024-03452-4

**Published:** 2024-06-08

**Authors:** Faisal AlNasser, Dara Entekhabi

**Affiliations:** Massachusetts Institute of Technology, Department of Civil and Environmental Engineering, Cambridge, MA 02139 USA

**Keywords:** Atmospheric dynamics, Environmental monitoring, Climate change

## Abstract

Airborne mineral dust significantly impacts air quality, human health, and the global climate. Due to sparse ground sensors, particularly in source regions, dust monitoring relies mainly on remote sensing through Aerosol Optical Depth (AOD) retrievals from polar-orbiting satellite optical instruments. These are valuable but lack the temporal resolution for precise plume tracking and source characterization. We introduce DustSCAN, a five-year, hourly dust plume dataset derived from the Spinning Enhanced Visible and InfraRed Imager (SEVIRI) images on geostationary-orbit Meteosat satellites. Using multi-channel infrared images, we detect atmospheric dust and track hourly dust-affected pixels. These are clustered into discrete plumes using the Density-Based Spatial Clustering of Applications with Noise (DBSCAN) algorithm. DustSCAN includes 9950 discrete plumes over 2018-2022 across the Sahara, the Arabian Desert, and Western and Central Asia. It complements existing resources and provides a framework for detailed analysis of dust sources, trajectories, and impacts. Its distinctive event-based and spatio-temporal detail offers an advancement in unraveling the complexities of dust storm dynamics.

## Background & Summary

### Monitoring Dust Plumes

Dust plumes originating from limited-extent arid and semi-arid regions can significantly impact the global climate and have both beneficial and detrimental effects. These plumes play a vital role in transporting nutrients across vast distances, thereby contributing to soil fertility in regions beyond their origin^1^. Dust plumes also pose a significant threat to human health and the economy. They significantly disrupt flight schedules and reduce solar panels’ efficiency, resulting in substantial operational challenges and economic losses^1,2^.

Dust sources are typically located in remote and arid areas, such as the Saharan Desert, which are difficult to equip with ground sensors^3^. Consequently, remote sensing data has become the most widely used resource to study atmospheric dust^4^. In particular, our understanding of dust storms has relied on Aerosol Optical Depth (AOD) estimates provided by low-earth orbiting satellites such as the Moderate Resolution Imaging Spectroradiometer (MODIS), the Total Ozone Mapping Spectrometer (TOMS), and the Multi-angle Imaging SpectroRadiometer (MISR)^4^. These estimates come with a significant shortcoming. Since they are based on a few daily passes, they do not offer continuous data on the evolution of dust plumes. Consequently, our understanding of dust plume sources, sinks, and pathways remains limited. Schepanski *et al*.^5^ compared identifying dust source areas from daily AOD frequencies with a backtracking method using quarter-hourly geostationary-orbit satellite images and found that the differences between the “back-tracking” and “frequency” relate to both temporal and spatial resolution. Low temporal resolution particularly limits plume tracking and source region identification.

### Utilizing Geostationary Orbit Satellites

Filling this gap in continuous monitoring can be achieved with instrument measurements on geostationary satellite platforms. The Spinning Enhanced Visible and InfraRed Imager (SEVIRI)^6^ on board the European Meteosat series of platforms is particularly noteworthy since its coverage encompasses the Sahara, the largest hot desert in the world.

In this contribution, the SEVIRI multichannel infrared measurements are used to develop the Dust RGB^7^, a false-color composite used to distinguish airborne dust. SEVIRI dust products have been a pivotal tool in dust research. They have been used as a visual verifier for the presence of extreme dust events^8–10^ and for the evaluation of dust prediction models^11^. Quantitatively, they have been utilized for mapping source areas and tracking plumes^5,12– 14^. Despite its extensive use and broad range of applications, public datasets of derived dust plumes are lacking.

Previous studies using SEVIRI to track dust plumes and identify source areas relied on imagery from Meteosat 8, positioned over 0^°^ longitude during 2002-2017. While the 0^°^ imagery is advantageous for observing the Sahara, it is significantly distorted for major deserts in Western Asia and the Arabian Peninsula. Furthermore, these studies faced some limitations. Spatially, some focused on a restricted area^12,15^. Temporally, certain analyses were confined to daytime observations, resulting in discontinuous tracking^16,17^. Additionally, the reliance on manual labeling has made scalability a challenge^14^.

To address these limitations and fill the data gap, we introduce the DustSCAN dataset. This dataset is generated through a semi-automated methodology that utilizes hourly SEVIRI images from the Indian Ocean Data Coverage (IODC), which started in 2017 after Meteosat 8 was repositioned to 41.5^°^E. This coverage encompasses a wide geographic area that includes the Sahara, Arabian Peninsula, and Western and Central Asia, thus observing most of the global “Dust Belt”. Our framework combines the use of the Dust RGB, machine learning, and subsequent manual quality control, providing extensive spatial coverage and round-the-clock tracking. We use the multi-year hourly dust fields in conjunction with a clustering algorithm to identify discrete dust plumes. The identification of events allows the determination of source areas, affected regions, and the extent of dust storm advection. In this data contribution, the discrete dust plumes based on SEVIRI hourly measurements during the 2018-2022 period are collected and shared on Figshare^18^. We also here report on the verification of these remote sensing-based dust plume fields using ground-based AERONET measurements of AOD.

## Methods

### Measurements Sources

**Fig. 1:**
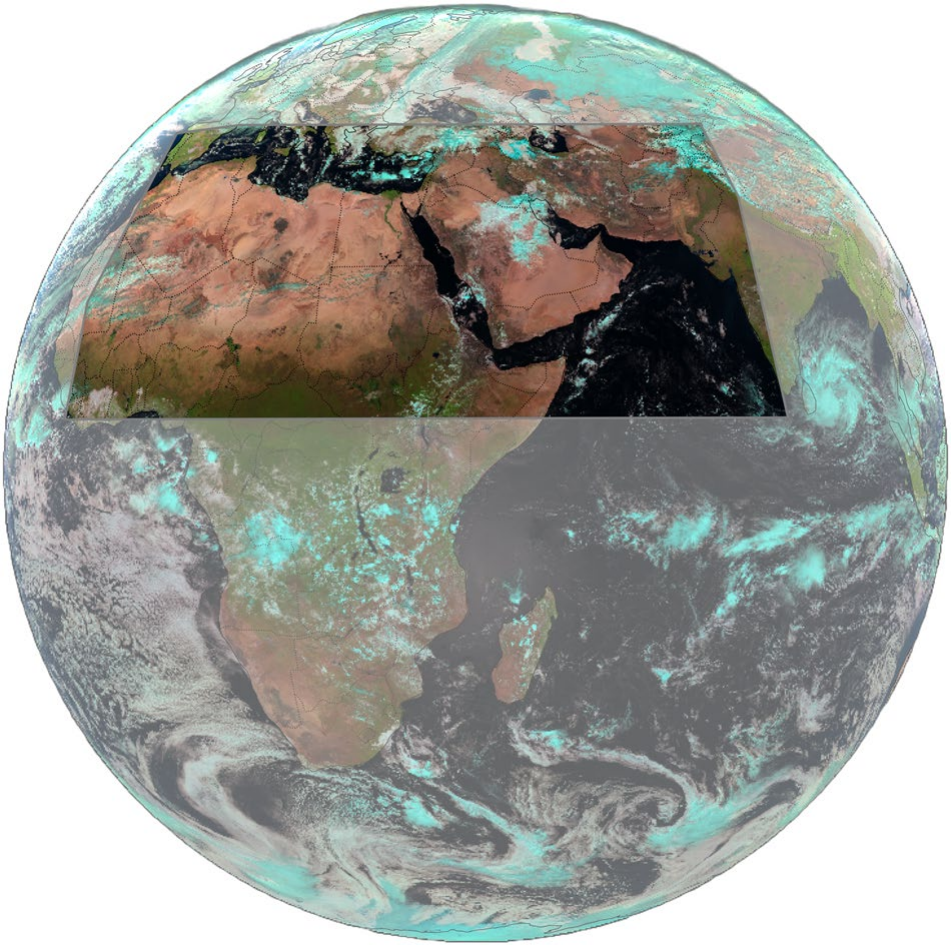
SEVIRI full disk true color RGB. The gray polygon highlights the area of interest for this study, covering most of the “Dust Belt” region. Clouds appear cyan in SEVIRI true color RGB.

This study uses data provided by the European Organisation for the Exploitation of Meteorological Satellites (EUMETSAT). Specifically, modified EUMETSAT Meteosat SEVIRI Level 1.5 imagery (2024) and Meteosat SEVIRI cloud mask data (2024)^19^. The SEVIRI data utilized is from the instrument’s observations in geostationary orbit over the Indian Ocean. Initially onboard Meteosat 8 at 41.5°E, and later onboard Meteosat 9 at 45.5°E. These satellite discs cover large portions of the Dust Belt, containing the world’s largest dust-emitting areas. Specifically, DustSCAN’s covered area stretches from 5.625° to 42.375° latitude and from -11.625° to 77.375° longitude, (Fig. 1). While the original images have a spatial resolution of 3 km at the nadir, they were resampled to 0.25 degrees for computational efficiency. The main parameters in this study are the brightness temperatures observed in the 12.0 *μ*m, 10.8 *μ*m, and 8.7 *μ*m bands, along with cloud masks.

### Dust Retrieval

**Fig. 2:**
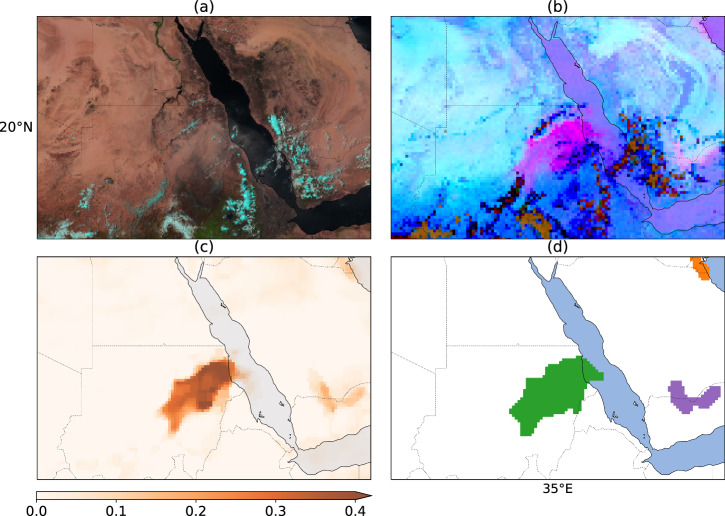
Dust plumes occurring on 2018-06-11, 11:00 UTC over Sudan, Rub’ al Khali, and Kuwait. (**a**) True color (clouds appear cyan in SEVIRI true color RGB). (**b**) Dust RGB (**c**) *PDI*_anomaly_ (**d**) Plume labels clustered by DBSCAN (each color represents a cluster. Refer to Fig. 3 for the same clusters in the spatio-temporal space).

To retrieve dust, we use the Dust RGB (Table 1 and Fig. 2(b)), a widely used false color Red-Green-Blue (RGB) composite that utilizes infrared band differences to isolate the signature of airborne dust^20^. In these false-color RGB images, dust appears magenta or pink, with thicker dust portraying a stronger magenta color^20^. The images are commonly used qualitatively by visual inspection. Here, an estimate of the amount of dust in a pixel is proposed by calculating the Euclidean distance from the pixel color to magenta, quantifying how strong the pink dust signal is, as described in the following equations:

1 $${P}_{{\rm{dist}}}=\sqrt{{(R-{R}_{{\rm{magenta}}})}^{2}+{(G-{G}_{{\rm{magenta}}})}^{2}+{(B-{B}_{{\rm{magenta}}})}^{2}}$$

2 $$PDI=\frac{Ma{x}_{{\rm{dist}}}-{P}_{{\rm{dist}}}}{Ma{x}_{{\rm{dist}}}}$$

**Table 1:** SEVIRI Dust RGB composite structure^7^.

Color Channel	SEVIRI Band	Range	Gamma
Red (R)	12.0 *μ*m – 10.8 *μ*m	− 4 to 2 K	1
Green (G)	10.8 *μ*m – 8.7 *μ*m	0 to 15 K	2.5
Blue (B)	10.8 *μ*m	261 to 289 K	1

Each color channel is scaled by the minimum and maximum values, and adjusted by a stretch factor, gamma, by raising it to the power of the inverse of the gamma value.

In (1), *P*_dist_ represents the distance in the color space to magenta. The variables *R*, *G*, and *B* represent a given pixel’s red, green, and blue color components, respectively. *R*_magenta_, *G*_magenta_, and *B*_magenta_ are the red, green, and blue color components of the color magenta, which correspond to [1,0,1] in RGB space. The Euclidean distance is calculated in the RGB color space between the given pixel’s color and magenta. Primarily in the remainder of this study, we use The Pink Dust Index (PDI) as in (2). PDI is derived from *P*_dist_ where it is normalized and inverted to be from 0 to 1 where higher values represent strong magenta (indicative of high dust concentration) and lower values represent weak magenta (indicative of low dust concentration). In this equation, *Max*_dist_ is the maximum possible Euclidean distance in the RGB color space. The diurnal variation in brightness temperatures can introduce a bias in PDI values. To ensure a more reliable measurement of dust concentration, we adopt a technique similar to the one employed by Ashpole and Washington^12^. This approach involves calculating an anomalous PDI as described in (3).

3 $$PD{I}_{{\rm{anomaly}}}=PD{I}_{{\rm{current}}}-PD{I}_{{\rm{month}}\_{\rm{hourly}}}$$

In (3), *PDI*_anomaly_ represents the “anomalously pink” measure, indicating the extent to which the current dust levels exceed the expected monthly norm for the given hour. The variable *PDI*_current_ denotes the PDI at the current time, while *PDI*_month_hourly_ refers to the cloud-screened monthly median PDI for the corresponding hour, over the same month. Fig. 2 includes an example of the *PDI*_anomaly_ metric along with true color and Dust RGBs. In this context, we utilize the *PDI*_anomaly_ as a qualitative metric indicating the presence of dust, similar to the approach adopted by Schepanski *et al*.^15^, as we note that this index may not consistently quantify the precise amount of dust loading due to potential variations in brightness temperature at different dust heights.

#### Limitations

The Dust RGB capitalizes on the thermal emissivity properties of fine, emitted dust particles, which differ from the hotter, coarser particles on the underlying desert surfaces. These differences give rise to dust appearing as magenta or pink^20,21^. Several factors influence the pronounced pink color of the dust, including skin temperature, humidity, dust altitude, and particle size distribution. In the Dust RGB, warmer surfaces appear blue, enhancing the contrast with the dust signal, whereas murky purple tones in colder conditions can obscure it, particularly in nighttime images. Additionally, the presence of water vapor can mask out the dust signal depending on the altitude, specifically, humidity weakens the dust signal at low altitudes (<1 km), whereas, at higher altitudes, dust becomes more apparent^21^. As a result, the retrievals are enhanced over deserts and weakened over vegetated surfaces^21^. Furthermore, smaller particle sizes tend to exhibit a pinker hue, enhancing the detectability of dust^22^. Lastly, dust plumes may be concealed beneath high clouds, rendering them unobservable.

### Plume Clustering

We isolate distinct plumes from the spatio-temporal *PDI*_anomaly_ array. In this approach, dust plumes are interpreted as moving clusters, described by Kalnis *et al*.^23^ as a set of objects that move close to each other for a long time interval, like migrating animals or a convoy of cars.

**Fig. 3:**
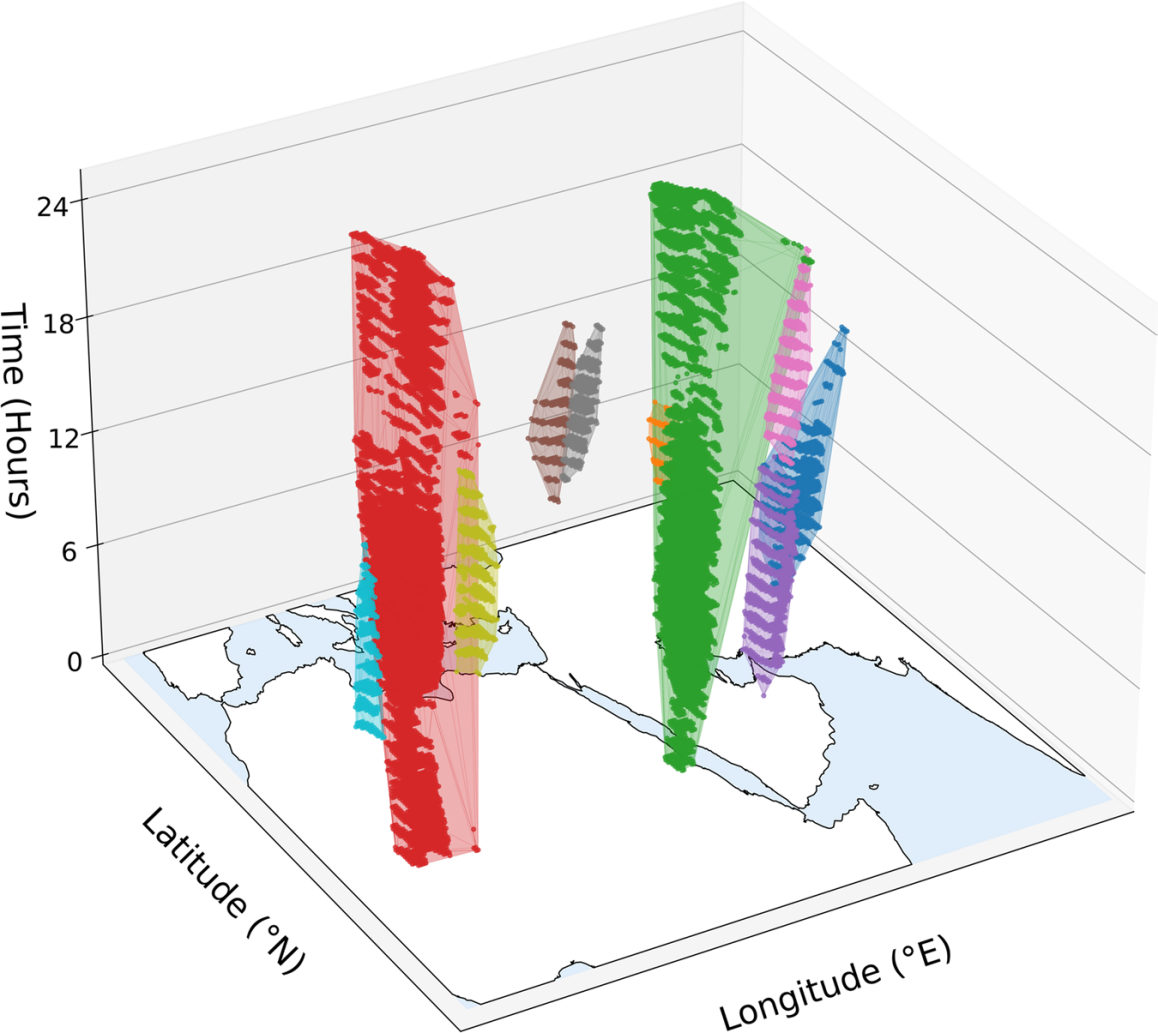
Plumes from 2018-06-11 as clustered by DBSCAN based on spatio-temporal connectivity of dust-affected pixels. Each color represents a cluster. Refer to Fig. 2(d) for a 2D spatial slice of the same data at time =11.

This approach is similar to spatial clustering but with a temporal dimension that enables tracking the movement of the spatial clusters with time. The identified clusters represent a plume traversing the data cube. This method operates on the assumption that distinct dust plumes are closely connected in space and time, which is a reasonable hypothesis as dust plumes mostly demonstrate clear start-to-end trajectories (see Fig. 3), unlike clouds which are mixed in the atmosphere.

Our choice of clustering algorithm is Density-Based Spatial Clustering of Applications with Noise (DBSCAN)^24^, a density-based clustering non-parametric algorithm that given a set of data points, it groups points that are closely packed together (points with many close neighbors) as clusters, and labels points that are in low-density regions (whose nearest neighbors are too far away) as outliers. DBSCAN is chosen based on three significant advantages:

1. The DBSCAN algorithm identifies clusters of arbitrary and irregularly shaped plumes within the data cube. This flexibility allows us to capture these events more accurately than with other methods that assume a specific shape for clusters.
2. Robustness against noise is another key advantage of DBSCAN. This is essential when dealing with satellite data, which often contains noisy points that obscure or distort clustering algorithms.
3. Lastly, DBSCAN does not require prior knowledge of the number of clusters, which is essential since the number of plumes in a given data cube is unknown beforehand. This allows the algorithm to organically determine the number of plumes from the data.

The progressive transition from Dust RGB to clustered plumes can be seen in Fig. 2(b–d). Figure 3 illustrates plume clusters in the spatio-temporal 3D space.

### Manual Quality Control

Retrieving dust plumes from Dust RGB imagery poses several challenges that require quality control and manual checking. These include:

1. Clouds: Certain clouds display a magenta signature in the Dust RGB, leading to spikes in the *PDI*_anomaly_. While such clouds are particularly prevalent along the coast of Morocco, as shown in Fig. 4(a,b), we have observed this effect in other regions as well. Ashpole and Washington^12^ excluded the Moroccan coastal area from their analysis. In this dataset, we recognize that this region is a significant pathway for dust to Spain and Portugal. Therefore, we manually remove the clouds by means of visual interpretation, not only from the Moroccan coast but from all affected regions, to ensure their effect did not skew the dataset. Additionally, dust plumes may get obscured by clouds and then reappear, potentially leading to their misidentification as new plumes. To address this issue, visual inspection is applied to merge such plumes.

**Fig. 4:**
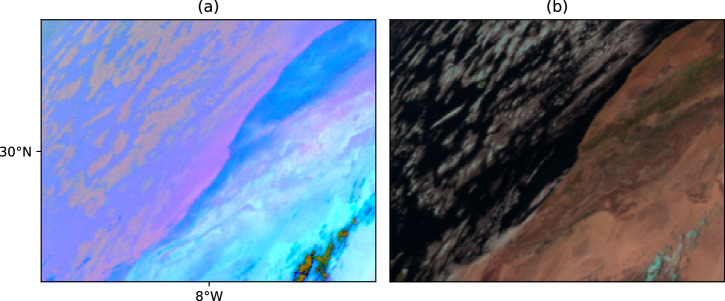
Certain clouds can resemble dust in Dust RGB imagery, necessitating manual validation to eliminate false positives. (**a**) Clouds on Morroco’s coast appearing magenta in the Dust RGB. (**b**) The same clouds in true color RGB.
2. Plume clustering: although plume clusters predominantly exist as separate entities in the spatiotemporal space (Fig. 3), there are instances where plumes intersect, especially during strong storms. This intersection can result in their being mis-clustered as a single plume. Ashpole and Washington^12^ addressed this issue by retaining the largest plume among the intersecting plumes. In this dataset, we depend on observer judgment to separate these intersecting plumes based on their trajectories and source area.
3. Corrupt data: some SEVIRI images are corrupt due to instrument technical issues, causing many errors in the process.
4. Night images: In the Dust RGB, the contrast between emitted dust and land diminishes during the night^5,21^. Although applying the hourly-based anomaly metric, *PDI*_anomaly_, partially compensates for these limitations, detection capabilities at night remain inferior to those during the day. However, manual adjustments can improve accuracy^5,25^. To do so, we carefully examine the progression of plumes in subsequent images to distinguish between stationary surface features and a passing dust plume.
5. Aerosol effects: The detection relies on changes in color by comparing them to a pristine sky reference, *PDI*_month_hourly_, where we assume cloud screening eliminates the effects of clouds and aerosols. However, persistent aerosol loadings in certain regions or months can invalidate this assumption. These effects reduce detection sensitivity to color variations, necessitating manual addition and extension of plumes.

Each of these challenges has been addressed in our dataset through stringent manual quality control. As a result, we have achieved a robust representation of dust plumes, ensuring the dataset’s reliability for further research.

## Data Records

The DustSCAN dataset contains 5 years (2018-2022) of hourly data at a 0.25-degree resolution and is geographically referenced to the World Geodetic System 1984 (WGS84). Each plume is assigned a unique number, allowing for consistent tracking and analysis of individual plumes over the span of five years. In addition, the dataset includes Dust RGB images for every hour, providing a visual representation of the dust conditions. Moreover, we incorporate EUMETSAT’s cloud mask and the solar zenith angle.

The dataset is hosted on Figshare^18^ and is divided into five files, one for each year (a year referenced in the file name spans from the beginning of December of the previous year to the end of November of the specified year), with each file approximately 16 GB in size. It is stored in the NetCDF file format and adheres to the NetCDF Climate and Forecast (CF) Metadata Conventions. The fields within each data file, and their respective dimensions, are as follows:

- Plume_ID: (time, latitude, longitude) - A unique identifier for plume clusters, clear pixels are assigned a value of 0.
- Dust_RGB: (time, latitude, longitude, band) - False-color Dust RGB image.
- PDI: (time, latitude, longitude) - Pink Dust Index, described in the methods section.
- Cloud_Mask: (time, latitude, longitude) - Cloud mask used to identify the presence or absence of clouds.
- Solar_Zenith_Angle: (time, latitude, longitude) - Solar zenith angle, derived from the SatPy^26^ library.
- Latitude: (latitude, longitude) - Latitude in degrees north.
- Longitude: (latitude, longitude) - Longitude in degrees east.
- Date: (time) - SEVIRI acquisition time In UTC time zone.

There are 43,824 hours from 2017/12/1 00:00 AM UTC to 2022/11/31 11:59 PM UTC, out of which, 43,546 are available in the dataset, and 295 hours are not available due to various technical issues, including instrument stoppages and server malfunctions.

### Supplemental Data

Understanding dust plume dynamics requires the integration of diverse datasets, some of which have been included for a comprehensive analysis. Namely, soil moisture data from the Soil Moisture Active Passive (SMAP)^27,28^ mission, Enhanced Vegetation Index (EVI) from MODIS^29^, and 10-meter wind vectors from the European Centre for Medium-Range Weather Forecasts (ECMWF) fifth-generation reanalysis ERA-5^30^. These datasets have been resampled, cropped to maintain consistent resolution and region, and added to the directory. Subsequently, plume properties, such as source area and duration, are extracted and integrated with the aforementioned datasets, providing a unified repository for the analysis of dust plumes. Details are provided in the usage notes.

## Technical Validation

### Dust Retrieval

To validate the dust retrieval, it is compared against measurements taken by the AErosol RObotic NETwork (AERONET)^31^, which consists of ground-based sun photometers that measure AOD. AERONET measurements are considered the ground truth reference for aerosol remote sensing and are used for validation and tuning^3,32–35^.

#### AERONET Data

For AOD (at 675 nm) and Angstrom Exponent (*α* at 440-870nm) retrievals, quality-assured Level-2 data was used. To keep dust-dominated AOD retrievals, Gkikas *et al*.^36^ have relied on *α* for aerosol characterization, associating the presence of mineral particles with low *α*. In line with their approach and recommended thresholds, we are keeping AOD records where the *α*_440−870*nm*_≤ 0.75. The validation encompasses sites situated within our study’s region, excluding those within 2 degrees of the boundary that are distant from source areas. To ensure a statistically significant and robust validation, only sites with over 30 days of measurements between December 2017 and November 2022 were considered, leading to the inclusion of 59 AERONET sites (Supplementary Table 1).

**Fig. 5:**
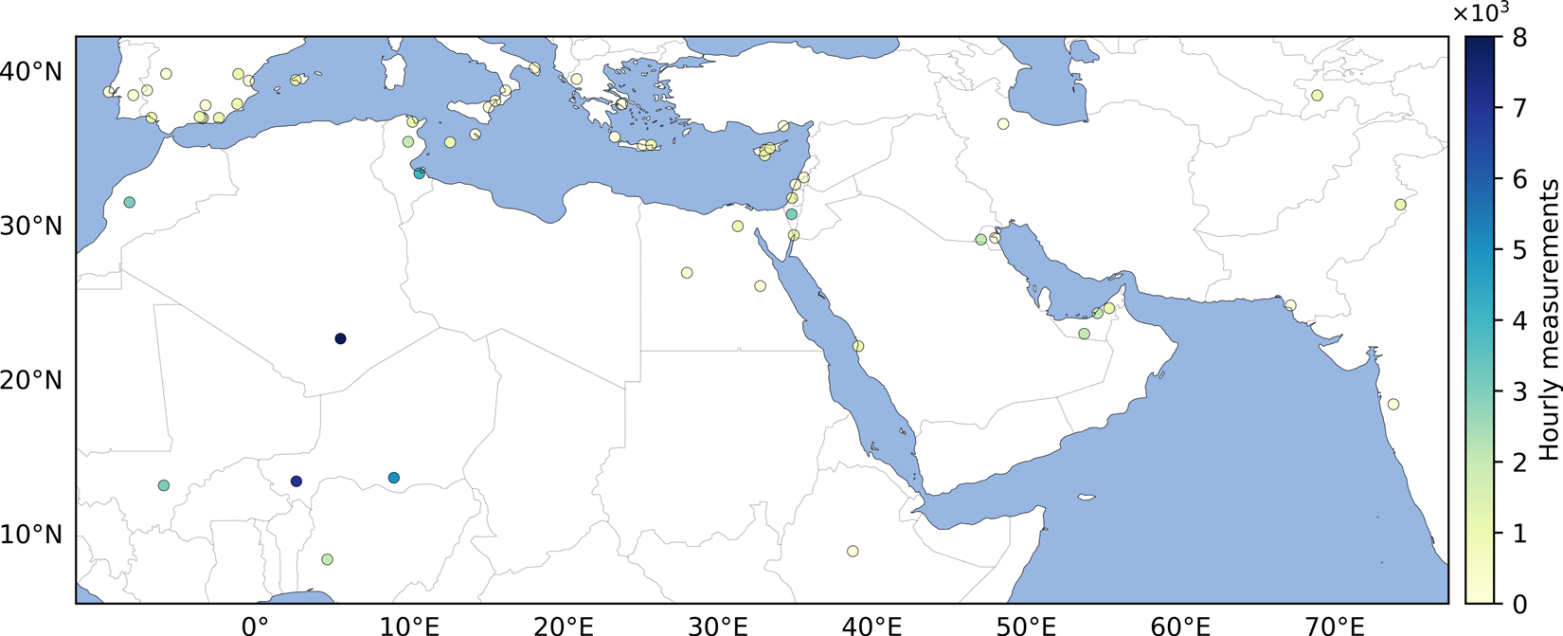
Number of hourly *AOD* measurements from AERONET sites used in the validation.

When analyzing a site, we examine the pixels within a 1.5-degree radius of the pixel closest to the site. If a dust plume from our data intersects this radius, the AOD for that hour is classified as “dust”. If no intersection occurs, the AOD is classified as “clear”, or it is excluded if the cloud mask indicates cloud presence. Additionally, to match the temporal resolution of DustSCAN, site AOD data is upsampled to an hourly frequency (Fig. 5 displays the number of hourly AOD measurements from each site).

#### Statistical Analysis

To evaluate the difference between dust-flagged and clear-flagged AOD measurements across all AERONET sites, denoted as *AOD*_*dust*_ and *AOD*_*clear*_. We perform a two-tailed independent two-sample t-test to determine the statistical significance of the observed difference between the means of the two groups, defined as:

4 $$t=\frac{{\overline{AOD}}_{{\rm{dust}}}-{\overline{AOD}}_{{\rm{clear}}}}{s\sqrt{\frac{1}{{n}_{{\rm{dust}}}}+\frac{1}{{n}_{{\rm{clear}}}}}}$$

**Fig. 6:**
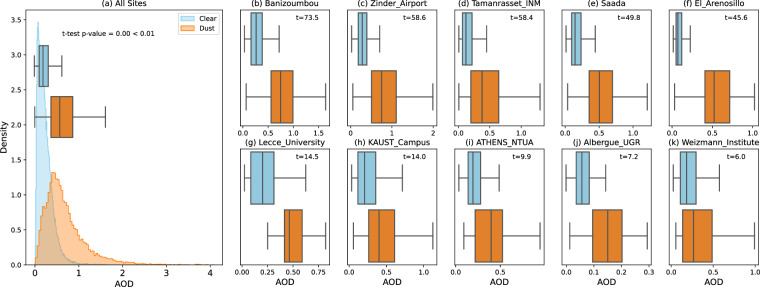
Validation of retrievals against AERONET. Where site measured Aerosol Optical Depth (AOD) at 675 nm is segmented to “dust” and “clear” based on the presence of dust in DustSCAN plumes. The x-axis represents AOD at 675 nm for all plots. (**a**) Aggregated AOD data from all AERONET sites, presented as a probability distribution with overlain summary statistics in a box plot. (**b**)-(**f**) Box plots of the 5 AERONET sites with the highest *t*_*stat*_. (**g**)-(**k**) Box plots for the 5 AERONET sites with the lowest *t*_*stat*_.

Here, ${\overline{AOD}}_{dust}$ and ${\overline{AOD}}_{clear}$ are the means of the two groups, and *s* is the pooled standard deviation. We confirm the significance of our validation with *t* = 194 and a *p* - *value* = 0.00 < 0.01, using aggregated data from all sites (Fig. 6(a)). This result was consistent across individual AERONET sites with sufficient data (*n* ≥ 30 in both groups) as shown in Fig. 6(b–k). This validates the robustness of our dust retrieval over AERONET sites.

### Plume Tracking

Validating dust plume tracking is challenging due to the lack of comparison datasets. In previous studies, this validation has been achieved through manual visual inspection of successive images and evaluation of plume movements^5,12,14^. As previously detailed, we adopt this approach, acknowledging the need for manual evaluation to ensure dataset accuracy.

#### Source area comparison

We assess plume tracking by backtracking the plumes to their source areas. This results in a source area map which can be compared to previous studies that similarly backtracked SEVIRI-derived plumes to find dust source areas. The main identified source areas (Figure 3 in AlNasser and Entekhabi^37^) are consistent with previous findings. Primarily, the Bodélé depression stands out as the most significant area^38^. Other identified sources are the lee side of the Aır Mountains^13,38,39^, Sudan^38^, the Syrian Desert^14^, Southern Iraq^14^, the Sistan Basin^14^ and the Thar Desert.

## Usage Notes

The provided code extracts from the dataset various properties of dust plumes and integrates them with supplementary data sources. Some of these properties have been previously described in dust plume literature, while others are newly introduced by us:

- Source area: Areas covered within the first hours.
- Source soil moisture: Soil moisture values co-located in time and space with the identified source area.
- Source wind speed: Wind speed values co-located in time and space with the identified source area.
- Source EVI: EVI values co-located in time and space with the identified source area.
- Center: The geometric centroid of the source.
- Coverage: All the areas covered by a plume.
- Extent: Euclidean distance from the center to the farthest point in the coverage. Highlights the distance of advection from the source in kilometers.
- Duration: Number of hours the plume lasts.
- Contribution: The total number of pixels emitted from a source. Used to highlight how much a source area contributes to dust emission.

This dataset has been employed in various analyses including mapping source areas^37^, finding affected regions, and studying emission co-factors such as soil moisture, wind speed, and vegetation cover.

### Supplementary information

Supplementary Table 1

## Data Availability

The code is available on github.com/faisalalnasser13/DustSCAN and is composed of Python Jupyter Notebooks.
